# The Role of Immune Cells and Cytokines in Intestinal Wound Healing

**DOI:** 10.3390/ijms20236097

**Published:** 2019-12-03

**Authors:** Xiang Xue, Daniel M. Falcon

**Affiliations:** Department of Biochemistry and Molecular Biology, University of New Mexico, Albuquerque, NM 87131, USA; DmFalcon@salud.unm.edu

**Keywords:** immune cells, cytokines, wound healing, intestine, inflammatory bowel disease

## Abstract

Intestinal wound healing is a complicated process that not only involves epithelial cells but also immune cells. In this brief review, we will focus on discussing the contribution and regulation of four major immune cell types (neutrophils, macrophages, regulatory T cells, and innate lymphoid cells) and four cytokines (interleukin-10, tumor necrosis factor alpha, interleukin-6, and interleukin-22) to the wound repair process in the gut. Better understanding of these immune factors will be important for developing novel targeted therapy.

## 1. Introduction

**Figure 1:**
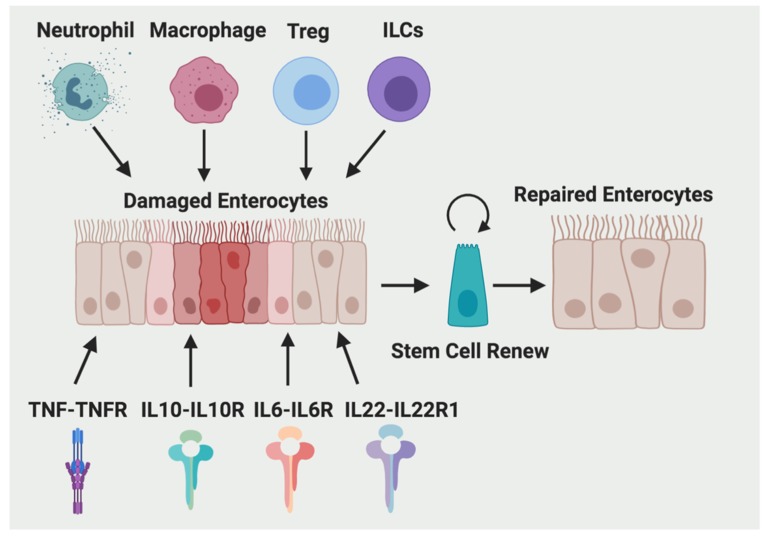
Immune cells and cytokines are contributing to intestinal wound repair. Four major immune cell types (neutrophils, macrophages, Treg) and ILCs), four cytokines (IL-10, TNF-α, IL-6, and IL-22) and their corresponding receptors are involved in stem cell renew and wound healing process in the intestine.

Wound healing in the intestine is a critical process affecting the prognosis of inflammatory bowel disease (IBD) [1]. Failure of healing could result in prolonged hospitalization, critical illness, and even death. Intestinal wound healing is consisted of three cellular events: restitution, proliferation, and differentiation of epithelial cells adjacent to the wounded area [2]. After intestinal tissue damage, the initial response is dominated by a proinflammatory type 1 immune response, whereas during the wound repair process, a more anti-inflammatory type 2 immune response will dominate to promote tissue regeneration and maintain tissue homeostasis [3]. A diverse array of evolutionarily ancient hematopoietic immune cell types, including lymphocytes, dendritic cells (DCs), monocytes, macrophages, and granulocytes, participate in this process. These immune cells secrete large amounts of cytokines and growth factors to signal to local tissue progenitors and stromal cells and promote wound repair. Here, we will discuss the contribution of four major immune cell types (neutrophils, macrophages, and regulatory T cells (Treg) and innate lymphoid cells (ILCs)) and four cytokines (interleukin-10 (IL-10), tumor necrosis factor alpha (TNF-α), IL-6, and IL-22) to the wound healing process in the intestine (Figure 1).

## 2. Immune Cells

The innate immunity is our first line of nonspecific and rapid defense against pathogens, whereas adaptive immunity confers specific long-lasting memory. Innate immune cells include neutrophils, macrophages, and DCs. The roles of neutrophils and macrophages in wound repair are discussed in detail below. DCs are antigen presenting cells mediating T cell activation and adaptive immunity, thus playing key roles in the crosstalk between innate and adaptive immunity [4].

### 2.1. Neutrophils

Neutrophils are the first responding leukocytes to sites of inflammation when the intestinal epithelial barrier is breached and the gut microbiota invade [5]. Mouse neutrophils migrate to wounded tissues begins 4 h and reach peak numbers 18 to 24 h after injury [6]. Neutrophils are short-lived cells with a half-life in the circulation of approximately 1.5 h and 19 h in mice and humans, respectively [7,8]. However, proinflammatory cytokines such as TNF-α, IL-1β, and IL-6 increase the lifespan of neutrophils [9], which may contribute to the resolution of inflammation [7].

#### 2.1.1. The Function of Neutrophils

**Figure 2:**
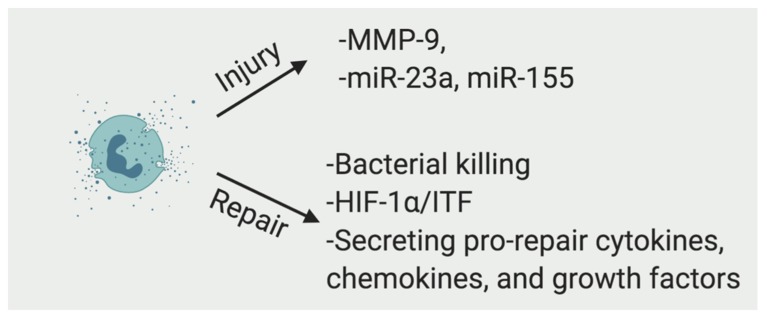
Neutrophils are a double edge sword in intestinal wound repair. Neutrophils damage intestinal mucosal through secreting MMP-9 and miRNA containing microparticles at acute phase of injury, but they can also promote wound repair through killing bacteria, modulating HIF-1α/ITF signaling and secreting pro-repair cytokines, chemokines, and growth factors.

The neutrophils can exert both destructive and protective effects in wound healing (Figure 2) [10]. Excess neutrophils in injured tissues impair healing and correlate with the crypt destruction and ulceration [11,12]. During intestinal inflammation, neutrophils undergo transepithelial migration and secrete a large amount of matrix metalloproteinase-9 (MMP-9) to disrupt epithelial intercellular adhesions, which leads to enhanced epithelial injury [13]. Neutrophil-derived miR-23a–and miR-155-containing microparticles also promote accumulation of double-strand breaks, which leads to impaired colonic healing [14].

As neutrophils have a key role in controlling microbial contamination and attracting monocytes and/or macrophages [15], individuals with too few neutrophils display not only higher risk for developing wound infections, but also delayed wound healing [16]. However, blocking neutrophil invasion or neutrophil depletion led to aggravated experimental colitis in animals, indicating a protective role of neutrophils in mucosal repair process [17].

Neutrophils kill bacteria through phagocytosis, neutrophils extracellular traps [18], antimicrobial peptides (including cathelicidins and β-defensins), microbicidal reactive oxygen species, and cytotoxic enzymes such as elastases, myeloperoxidase, and MMPs [19]. Infiltrating neutrophils deplete local oxygen to stabilize the transcription factor hypoxia inducible factor (HIF)-1α in wounded human and murine intestinal mucosa and promote resolution of inflammation. HIF-1α stabilization also protects barrier function through induction of intestinal trefoil factor (ITF) [20,21]. It has been shown that the probiotic *Lactobacillus rhamnosus* GG restored alcohol-reduced ITF in a HIF dependent manner [22].

In addition to eliminating bacteria and adjusting the wound microenvironment through oxygen metabolism, neutrophils promote wound repair by secreting pro-repair cytokines, chemokines, and growth factors. After dextran sodium sulfate (DSS)-induced mucosal injury, neutrophil-derived transforming growth factor-beta (TGF-β) activates MEK1/2 signaling and induces the production of the EGF-like molecule amphiregulin (AREG) in intestinal epithelial cells, which protects intestinal epithelial barrier function and ameliorates DSS-induced colitis [23].

#### 2.1.2. The Regulation of Neutrophils

Antibiotic treatment of dams reduced circulating and bone marrow neutrophils via reducing IL-17-producing cells in the intestine and their production of granulocyte colony-stimulating factor (G-CSF) [24]. In contrast to the mucosal protective effects of acute HIF-1α activation described above, we have previously showed that chronic activation of epithelial HIF-2α increased the proinflammatory response [25] and cancer development [26,27]. Among various mechanisms, HIF-2α can directly regulate the expression of neutrophil chemokine CXCL1, which facilitates the recruitment of neutrophils in colitis associated colon tumor [28]. Similarly, during intestinal inflammation, the intestinal epithelial production of neutrophil chemotactic cytokine IL-8 (chemokine C-X-C motif ligand 8, CXCL8) is increased by proinflammatory cytokines IL-1β, TNF-α, or interferon-γ (IFN-γ) [29]. A recent report also showed that IFN-γ induced expression of a neutrophil ligand intercellular adhesion molecule-1 (ICAM-1) on the intestinal epithelium apical membrane, which led to enhanced epithelial permeability and facilitated neutrophil transepithelial migration [30]. Interestingly, the enhanced ICAM-1 and neutrophil binding results in decreased neutrophil apoptosis, activation of Akt and β-catenin signaling, increased epithelial cell proliferation, and wound repair [31]. Il-23 signaling is also required for maximal neutrophil recruitment after DSS treatment [32].

### 2.2. Macrophages

Intestine contains the largest pool of macrophages in the body [33]. It was long considered that, different from other tissues, embryonic-derived macrophages only populate the colon during neonatal stage. Ly6C (hi) circulating monocytes that recruited and differentiated locally into anti-inflammatory macrophages gradually replace embryonic macrophages at the time of weaning. However, a recent study found that there are three subpopulations of macrophage in the mouse gut: Tim-4+CD4+ macrophages are locally maintained, whereas Tim4-CD4+ and Tim4-CD4− macrophages are replenished from blood monocytes [34]. Another study showed that a population of self-maintaining macrophages aroused from embryonic precursors and bone marrow derived monocytes persists in the intestine throughout adulthood. Deficiency of this population leads to vascular leakage, reduced intestinal secretion and motility [35]. In mice, colonic macrophages are identified by the following marker expression profile: CX3CR1int/hi CD64+ CD11b+ CD11clo/int F4/80+ Ly6C-/lo MHCII+ CD172α+ CD103− SiglecF− CCR7− [36,37]. The lifespan of macrophages is at least 1–2 week [36,38].

#### 2.2.1. The Function of Macrophages

Defects in macrophage differentiation may contribute to increased susceptibility to IBD [39]. Compared with blood monocytes, human intestinal macrophages display downregulated cytokine production upon bacterial products stimulation but preserve phagocytic and bactericidal activity [40]. Thus, intestinal macrophages (CX3CR1 hi) normally possess an anti-inflammatory phenotype during homeostasis via constitutive production of IL-10 [41], whereas Toll-like receptor-responsive proinflammatory macrophages accumulate in the colon and may contribute to disease severity and progression in IBD [37]. However, colonic anti-inflammatory macrophages are still present and promote tissue repair after injury [42]. Studies in mice lacking macrophages suggested that macrophages are necessary for proper epithelial regeneration after DSS injury [43]. Furthermore, Trem2 expressing macrophages are required for efficient mucosal regeneration after colonic biopsy injury [44]. In addition, macrophage-secreted WNT ligands enhance intestinal regeneration response against radiation [45]. Transfer of anti-inflammatory macrophages accelerate mucosal repair in 2, 4, 6-trinitrobenzenesulfonic acid (TNBS)-treated mice through the activation of the Wnt signaling pathway [46].

#### 2.2.2. The Regulation of Macrophages

Macrophage-dependent wound repair in response to DSS-induced colonic injury is markedly diminished in germ-free mice, indicating an essential role of microbiota in macrophage-mediated wound healing [43]. Commensal microbiota-derived local signals in the intestine are essential for recruiting macrophages from circulating monocytes [33]. Breeding of mice in germ-free conditions had a detrimental effect on the number of mature macrophages populating the adult colon compared to mice house in conventional conditions.

However, the small intestine macrophages are regulated by dietary amino acids but not microbiota [47]. Mice fed a protein-free diet had significantly lower levels of IL-10-producing macrophages but not IL-10-producing CD4+ T cells in their small intestine, compared with control-diet fed mice [47]. Depletion of commensal bacteria did not affect numbers of mature macrophages in the small intestine, spleen, or bone marrow, indicating that the recruitment of macrophages to the small intestine is regulated independently of the microbiota [47]. Depletion of microbiota also has no effect on the repair of small intestinal injury [48].

### 2.3. Regulatory T Cells (Treg)

Treg cells are a subset of CD4+ T cells that can inhibit T helper (Th) cells through the release of anti-inflammatory cytokines, such as IL-10 and TGF-β, or by direct contact with Th cells [49]. Th1 cells are induced by IL-12 and secrets IFN-γ, whereas Th2 cells are induced by IL-4 and releases IL-5 and IL-13 [50]. Crohn’s disease (CD) has been long considered to be driven by a Th1 response, whereas the notion that UC is mediated by Th2 response is still controversial [50]. There are two best-characterized subsets of Treg cells that suppress the immune response: forkhead box P3+ (Foxp3+)-positive Treg cells and Foxp3-negative type 1 Treg (Tr1) cells [51]. Foxp3+ Tregs are mainly derived from the thymus, and some travel to the intestine where they inhibit inappropriate immune reactions. Tregs are significantly reduced in peripheral blood and colonic mucosa of IBD patients [52].

#### 2.3.1. The Function of Treg

Foxp3+Tregs promotes the healing of UC through endogenous vascular endothelial growth factor receptor 1 tyrosine kinase (VEGFR1-TK) signaling as mucosal repair of DSS-induced colitis is delayed in VEGFR1-TK knockout mice [53]. For Tr1 cells, in addition to secreting immunosuppressive cytokines IL-10 and TGF-β [54], they secrete IL-22 to regulate repair of the epithelium and protect barrier function of human intestinal epithelial cells [55]. It has been shown recently that patients with refractory in CD were well tolerated with ovalbumin-specific Tr1-based therapy and had a dose-related efficacy [56].

#### 2.3.2. The Regulation of Treg

The microbiota affects the frequency and function of mucosal Tregs. The frequency of Tregs increased in the colon and lamina propria of the small intestine after weaning suggesting a role of the microbiota [57]. Post-weaning accumulation of Tregs was impaired in germ-free or antibiotic-treated mice compared with conventionally housed mice. In addition, germ-free mice fed fecal suspensions from conventionally housed mice saw a substantial increase in Treg levels. Indigenous Clostridium species were reported to play a central role in the induction of IL-10 producing Foxp3+ Tregs in the colon and small intestine in mice [57]. Additionally, it appears as though Clostridia bacteria have a direct role in modulating immune cell populations in the gut as many Clostridium-colonized mice were observed to have Tregs negative for Helios—a transcription factor reported to be expressed in thymus-derived “natural” Tregs. Therefore, the absence of Helios suggests that increasing levels of Tregs in the colon may be induced Treg (iTregs). Indeed, the culture of splenic CD4+ cells in the presence of supernatant of intestinal epithelial cells from Clostridium-colonized mice induced the differentiation of FoxP3-expressing cells. Furthermore, this effect was diminished by neutralizing antibody against TGF-β. Interestingly, it appears that iTregs also play a role in maintaining gut homeostasis as demonstrated in a DSS treatment model of colitis. Symptoms of colitis, such as weight loss, rectal bleeding, colon shortening, edema, mucosal erosion, crypt loss, and cellular infiltration, were all reduced in Clostridium-colonized mice treated with DSS compared to controls.

Different from microbiota-induced Treg cells, dietary antigens from solid food induce the main part of the short-lived small intestinal periphery Treg cells [58].

### 2.4. Innate Lymphoid Cells (ILCs)

**Figure 3:**
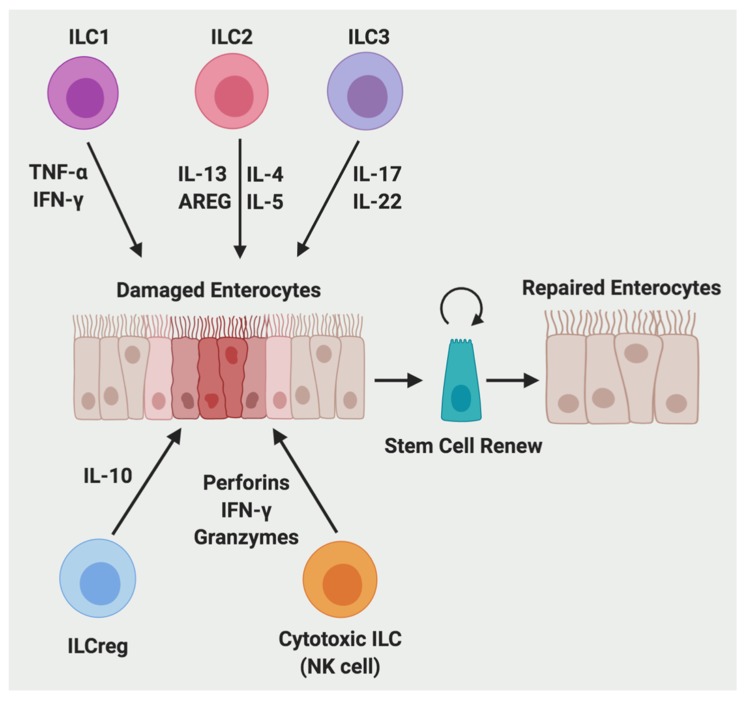
ILCs contribute to the process of intestinal wound repair. During wound healing, ILC1 secretes TNF-α and IFN-γ; ILC2 secretes IL-4, IL-5, IL-13, and AREG; ILC3 secretes IL-17 and IL-22; ILCreg secretes IL-10; and cytotoxic ILC (NK cell) secrete perforins, IFN-γ, and granzymes.

ILCs are mainly tissue-resident lymphocytes that lack adaptive antigen receptors expressed on T cells and B cells (Figure 3) [59]. They are generally classified into three subgroups according to their cytokine and transcription factor expression, which parallel with adaptive CD4+ Th cell subsets: group 1 (ILC1), group 2 (ILC2), and group 3 (ILC3) [59,60,61]. ILC1s are dependent on the T-box transcription factor (T-bet) for their development and function, and they produce IFN-γ and TNF-α [62]. ILC2s are dependent on GATA binding protein 3 (GATA3) and RAR-related orphan receptor alpha (RORα) [63], and produce type 2 cytokines, including IL-4, IL-5, IL-9, and IL-13 [64]. ILC3s are dependent on the transcription factor RAR-related orphan receptor gamma (RORγt) and can produce IL-17 and/or IL-22 [59,65]. ILC1s react to intracellular pathogens, such as viruses and tumors; ILC2s respond to large extracellular parasites and allergens; and ILC3s combat extracellular microbes, such as bacteria and fungi [59]. In addition, a recent report identified a regulatory subpopulation of ILCs (called ILCregs) that exists in the mouse and human gut and Id3 is a fate decision marker for their development [66]. Compared with these ILC subsets, conventional natural killer (NK) cells has a similar developmental process and quick effector functions, thus NK cells are defined as cytotoxic ILCs, which parallel with adaptive CD8+ cytotoxic T lymphocytes [61]. Mature NK cells are dependent on the transcription factor eomesodermin (Eomes), and produce perforins, IFNγ, and granzymes [67]. NK cells control certain viruses such as herpesviruses and cytomegalovirus and tumors [68].

#### 2.4.1. The Function of ILCs

ILCs maintain tissue homeostasis but also contribute to inflammatory diseases including IBD [69]. ILCs promote the resolution of inflammation and tissue repair [70].

ILC1s have a crucial role in promoting innate immunity to intracellular pathogens, such as T. gondii, by secreting TNF-α and IFN-γ to recruit inflammatory myeloid cells [70]. Intraepithelial ILC1s expand in CD patients and depletion of intraepithelial ILC1s reduced proximal colon inflammation in the anti-CD40-induced colitis model in mice [71].

ILC2s rapidly respond to helminth parasite infection [70]. ILC2s are increased in patients with ulcerative colitis (UC) and play an important role in the tissue reparative response [72]. ILC2s secreted IL-13 binds with its receptor IL-13Rα1 and activates transcription factor Foxp1 to promote β-catenin pathway-dependent intestinal stem cell renewal [73]. In addition, IL-33 can stimulate ILC2s to produce AREG in the colon and promote intestinal epithelial cell regeneration in a model of DSS-induced colitis [74].

ILC3s promotes innate immunity to extracellular bacteria and fungi, such as *Citrobacter rodentium* and *Candida albicans* [70]. ILC3s are decreased in inflamed tissue in both CD and UC patients [72] and are required for tissue repair and regeneration in the inflamed intestine [75]. Adherent CD-associated microbiota induces the CX3CR1+ mononuclear phagocyte-derived TNF-like ligand 1A (TL1A) [76], which stimulates the production of ILC3-derived IL-22 and increases mucosal healing in human IBD [77]. ILC3s are the main source of intestinal IL-22 and the symbiotic commensal microbiota represses this IL-22 production via inducing epithelial expression of IL-25 [75]. In graft versus host disease, radioresistant ILC3s-produced IL-22 protects intestinal stem cells from immune-mediated tissue damage [78]. Mechanistically, IL-22 activates signal transducer and activator of transcription 3 (STAT3) signaling to increase antiapoptotic proliferative response in Lgr5+ stem cells, promoting epithelial regeneration and reducing intestinal pathology and mortality from graft-versus-host disease [79]. Moreover, dietary aryl hydrocarbon receptor aryl hydrocarbon receptor (Ahr) ligands such as glucosinolates promote IL-22 production from ILC3s and protect intestinal stem cells against genotoxic stress [80]. In addition, ILC3s produced IL-22 production also protects damage to the intestine induced by infection and chemotherapy [81,82]. Apart from IL-22, ILC3s secreted IL-17 and IFN-γ is dependent on IL-23 stimulation and is required in *Helicobacter hepaticus*-mediated innate colitis [83].

ILCregs suppress the activation of ILC1s and ILC3s via secretion of IL-10 and promote innate intestinal inflammation resolution induced by several inflammatory stimuli including DSS, anti-CD40 antibody, *Salmonella typhimurium*, and *Citrobacter rodentium* in Rag1 −/− mice [59].

NK cells with cytolytic potential are accumulated in colonic lamina propria of individuals with active IBD [84], and thiopurines can normalize NK cell numbers by inhibition of Rac1 activity to induce apoptosis [85]. Activated NK cells produce proinflammatory cytokines such as IFN-γ and TNF-α to augment CD4+ T cell proliferation and Th17 differentiation, which contributes to exacerbated inflammatory response [86].

#### 2.4.2. The Regulation of ILCs

Commensal microbiota regulates the transcriptional gene expression and epigenetic regulation in ILCs [87]. RNA- and ATAC-seq integration identified that c-MAF and BCL6 regulate the plasticity between ILC1 and ILC3 in the intestine [69]. Moreover, the Ahr signaling is critical in regulating intestinal ILC2-ILC3 balance. This was demonstrated by the fact that Ahr knockout mice have altered gut ILC2 transcription with increased expression of anti-helminth cytokines such as IL-5 and IL-13, whereas Ahr activation increases gut ILC3 to better control *Citrobacter rodentium* infection [88]. Furthermore, ILC1 and ILC3 undergo retinoic-acid dependent upregulation of gut homing receptors CCR9 and α4β7, while ILC2 acquire these receptors during development in the bone marrow [89]. These gut homing receptors are also critical for optimal control of *Citrobacter rodentium* infection. For ILCregs, autocrine TGF-β1 is critical for their expansion during inflammation [66]. NK cells are regulated by various cytokines such as type I IFN, IL-12, IL-18, IL-15, IL-2, and TGF-β1 [90].

## 3. Cytokines

### 3.1. IL-10

#### 3.1.1. The Source of IL-10

IL-10 production in the colon was mainly from lamina propria macrophage and regulatory T cells [91]. Macrophage-specific knockout of IL-10 had a detrimental effect on intestinal wound healing using a colon biopsy-induced injury model in vivo indicating macrophages are an important source of IL-10 [92]. In addition, intestinal epithelial cells and Th1 cells are also able to produce IL-10 [93,94].

#### 3.1.2. The Function of IL-10

Analysis of biopsy-induced murine colonic wounds revealed an increase in IL-10 as soon as 24 h post-injury suggesting an upregulation during intestinal wound repair [92]. Exposure of intestinal epithelial cells to recombinant IL-10 was demonstrated to enhance wound repair in vitro whereas knockdown of IL-10 receptor ameliorated this effect. IL-10 promotes epithelial activation of cAMP response element-binding protein (CREB) and secretion of pro-repair WNT1-inducible signaling protein 1.

In a mouse model of small intestine epithelial injury induced by Indomethacin, MHC-II^+^ CD64^+^ Ly6C^+^ macrophage-derived IL-10 produced during the acute phase of injury was demonstrated to be critical for wound recovery [48].

#### 3.1.3. The Regulation of IL-10

Macrophage- and regulatory T cell-derived IL-10 production was demonstrated to be microbiota-dependent in the colon, as germ-free mice responded to LPS-stimulation by producing more TNF-α and IL-6 but less IL-10 [91]. In Th1 cells, microbiota-derived short-chain fatty acids promote IL-10 production via G-protein coupled receptors 43/B lymphocyte induced maturation protein 1 signaling [94].

### 3.2. TNF-α

TNF-α, also known as TNF, was first identified as a tumoricidal protein that mediates endotoxin-induced hemorrhagic necrosis in sarcoma and other transplanted tumors in 1975 [95]. Later in 1984 human TNF was cloned [96].

#### 3.2.1. The Source of TNF-α

TNF is produced predominantly by activated macrophages and T lymphocytes as a plasma membrane bound 26 kDa precursor glycoprotein. TNF-α converting enzyme (TACE; also known as ADAM-17) mediates the cleavage in the extracellular domain of TNF-α precursor and releases a soluble 17 kDA form [97]. In addition to macrophages and T lymphocytes lineage, a wide range of cells can produce TNF-α, including mast cells, B lymphocytes, natural killer (NK) cells, neutrophils, endothelial cells, intestinal epithelial cells (IECs), smooth and cardiac muscle cells, fibroblasts, and osteoclasts [98,99]. TNF-α is not usually detectable in healthy individuals, but elevated serum and tissue levels are found in inflammatory conditions [100], and serum levels correlate with the severity of infections [101,102].

#### 3.2.2. The Function of TNF-α

TNF-α is a key regulator of inflammation and has been involved in many human diseases, including psoriasis, rheumatoid arthritis, and IBD [103]. Anti-TNF-α therapy is the best available therapeutic option to induce mucosal repair and clinical remission in IBD patients [104]. However, a recent report showed that TNF-α blockage may cause dysbiosis and increased Th17 cell population in the colon of healthy mice [104]. Another report demonstrated that TNF-α promotes colonic mucosal repair through induction of the platelet activating factor receptor (PAFR) via NF-B signaling in the intestinal epithelium. Increased PAFR expression leads to activation of epidermal growth factor receptor Src as well as increased Rac1 and FAK signaling to promote cellular migration and wound closure. Consistently, TNF-α neutralization ablates PAFR upregulation and impairs intestinal wound repair [105]. In addition, bone marrow-derived TNF-α binds to epithelial TNF receptors (TNFRs) and activates epithelial beta-catenin signaling, promotes intestinal stem cell proliferation and IEC expansion, and helps mucosal healing in chronic colitis patients [98]. This was shown as enhanced apoptosis, reduced IEC proliferation, and decreased Wnt signaling when stimulated with anti-CD3 mAb in TNF-deficient (Tnf −/−) mice [76]. TNFR2 was increased in the epithelial cells from IBD patients and disruption of TNFR2 in naïve CD8+ T cells increased the severity of colitis in Rag 2 −/− mice [106,107]. TNF-induced intestinal NF-κB activation is also crucial for prevention of local intestinal injury following ischemia–reperfusion [108].

#### 3.2.3. The Regulation of TNF-α

At the transcriptional level, the TNF gene is induced in response to a diversity of specific stimuli including inflammation, infection, and stress [109]. Bacterial endotoxin specially activates TNF-α gene expression [110]. Analysis of human TNF-α promoter indicated that transcription factors such as Ets and c-Jun are involved in the transcriptional regulation of TNF-α [111]. Previously, we have also shown that HIF-2α is a positive regulator of TNF-α production in the intestinal epithelium [25].

### 3.3. IL-6

The IL-6 family of cytokines include IL-6, IL-11, IL-27, IL-31, oncostatin M, leukaemia inhibitory factor, ciliary neurotrophic factor, cardiotrophin 1, and cardiotrophin- like cytokine factor 1 [112]. They play crucial roles in cell proliferation, survival, migration, invasion, and inflammation [113].

#### 3.3.1. The Source of IL-6

IL-6 is mainly produced by lymphocytes, myeloid cells, fibroblasts and epithelial cells [114]. Enterocyte IL-6 production is increased during inflammatory conditions such as sepsis and endotoxemia [115].

#### 3.3.2. The Function of IL-6

IL-6 and its soluble receptor s-IL6R are highly elevated in the colonic mucosa of IBD [116]. The single nucleotide polymorphism rs2228145 in IL-6R associates with increased levels of s-IL6R, as well as reduced IL-6R signaling and risk of IBD [117]. A randomized clinic trial in 36 patients with active CD showed that 80% of the patients given a human anti-IL-6R monoclonal antibody biweekly at a dose of 8 mg/kg had a clinical response compared with only 31% of placebo injected patients, indicating that targeting IL-6 signaling may serve as a promising strategy for CD [118].

IL-6 promotes IEC proliferation and regeneration, and IL6-deficient mice exhibit elevated IEC apoptosis following exposure with DSS [119]. The proliferative and antiapoptotic effects of IL-6 are mainly mediated by the transcription factor STAT3, whose IEC-specific ablation leads to more severe DSS-induced colitis compared to wild-type mice [98]. In addition, the IL-6 co-receptor gp130 stimulates intestinal epithelial cell proliferation through Yes-associated protein (YAP) and Notch signaling, which leads to aberrant differentiation and promotion of mucosal regeneration [120]. Activation of YAP [121] and Notch [122] are required for mucosal regeneration after DSS challenge.

#### 3.3.3. The Regulation of IL-6

IL-6 is a multifunctional NF-kB-regulated cytokine that acts on epithelial and immune cells [119]. Endotoxin and IL1-β stimulates IL-6 production from IEC [123]. IFN-γ alone did not stimulate but synergistically potentiated the effect of IL-1β stimulated IL-6 production [124]. In contrast, TGF-β and cAMP were found to enhance IL-6 secretion, and they could potentiate IL-1β stimulated IL-6 production [125,126]. All four major transcription factors, i.e., NF-B, activator protein-1, CCAAT/enhancer binding protein, and CREB, are involved in the cAMP activated IL-6 production [126].

### 3.4. IL-22

IL-22, a cytokine of the IL-10 superfamily, was originally identified as an IL-9-induced gene in mouse T cells, and was named as IL-10-related T cell-derived inducible factor as it shares 22% amino acid identity with IL-10 [127]. IL-22 binds to a functional receptor complex composed of two chains: IL-22 receptor 1 (IL-22R1) and IL-10R2 [128].

#### 3.4.1. The Source of IL-22

IL-22 is produced from many different cell types such as activated T, NK cells and CD11c+ cells [129,130,131]. As mentioned above, in the intestine ILC3s are the main source of IL-22 [75].

#### 3.4.2. The Function of IL-22

IL-22 is increased in the intestine in patients with IBD as well as murine DSS colitis [132,133,134,135]. Although IL-22 increases the gene expression of other proinflammatory cytokines, such as IL-8 and TNF-α in intestinal epithelial cells, IL-22 promotes wound healing of the intestinal epithelium in vitro through stimulation of cell migration via phosphatidylinsitol 3-kinase signaling and beta-defensin-2 expression [135]. In addition, as mentioned above, IL-22 protects intestinal stem cells in graft versus host disease via activating STAT3 signaling and protects against genotoxic stress [78,79,80]. IL-22 knockout mice showed delayed recovery from biopsy forceps and DSS induced mucosal injury [129,130]. Due to decreased production of antimicrobial proteins, such as RegIIIβ and RegIIIγ, IL-22 knockout mice have increased susceptibility to *Citrobacter rodentium* infection [134]. A recent study showed that IL-22 induces expression of H19 long noncoding RNA in epithelial cells to promote epithelial proliferation and mucosal regeneration [136]. Exogenous IL-22 also mitigates *Citrobacter rodentium* infection mediated colitis in mice with depletion of CX3CR1+ mononuclear phagocytes [77]. Local gene delivery of IL-22 into the colon promotes recovery from acute intestinal injury via STAT3 mediated mucus production [137].

#### 3.4.3. The Regulation of IL-22

Human intestinal ILC3 production of IL-22 is regulated by microbial stimulated IL-23 and IL-1β from CX3CR1+ mononuclear phagocytes [77]. IL-22 can be neutralized by its soluble receptor IL-22 binding protein (IL-22BP; also known as IL-22RA2), which specifically binds IL-22 and prevents its binding with membrane-bound IL-22R1 [138]. IL-22 is most highly expressed at the peak of DSS and biopsy induced intestinal tissue damage, whereas IL-22BP has the lowest expression at this time [139]. AhR also increases IL-22 production to protect against trinitrobenzene sulfonic acid-induced colitis [140]. A recent report showed that the receptor-interacting protein kinase 3 promotes intestinal tissue repair after DSS colitis via induction of IL-22 expression in a IL-23 and IL-1β dependent manner [141].

## 4. Concluding Remarks and Perspectives

In conclusion, inflammatory cells and cytokines play critical roles in intestinal tissue repair. The introduction of anti-TNF-α antibodies has already been a great advance for IBD targeted therapy. Thus, targeting the above cells and cytokines may represent novel therapies for IBD. A recent phase II clinical trial showed that a human blocking antibody against T cell and NK cell receptor natural killer group 2D induced significant clinical remission in active CD patients after 12 weeks [142].

This review only covered some of the most important immune cell types and cytokines; others may also play an important role in wound healing. For example, IL-36γ was induced during experimental colitis and human IBD in a microbiota-dependent manner [143]. IL-36R-deficient mice showed delayed recovery after DSS-induced intestinal injury with profound IL-22 reduction and impaired neutrophil accumulation. In addition, we did provide much detail about the interaction between different cell types; for example, inflammatory monocytes may inhibit neutrophil activation in a prostaglandin E2 dependent manner [144]. Also, the bidirectional interactions between macrophages and lymphocytes were previously reviewed [145].

**Figure 4:**
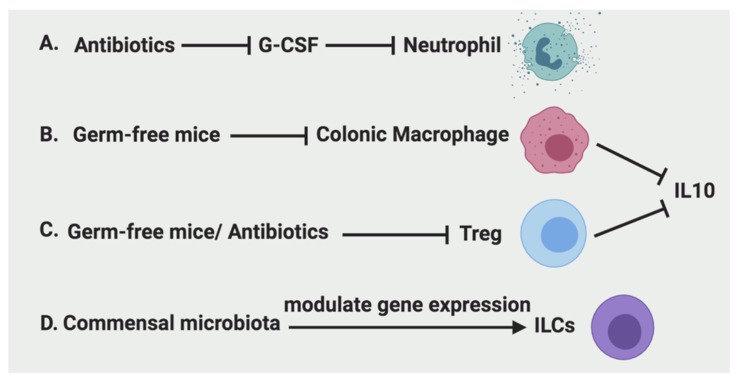
Regulation of immune cells by microbiota. A. Antibiotics can inhibit the recruitment of neutrophils by inhibition of G-CSF production. B. Colonic macrophages and their secretion of IL-10 are significantly reduced in germ-free mice. C. Tregs and their production of IL-10 are reduced in germ-free mice as well as antibiotics treatment. D. The gene expression of ILCs are modulated by commensal microbiota.

As discussed above, microbiota is essential in regulating neutrophil recruitment, colonic macrophage development, Treg function, and gene expression of ILCs (Figure 4). Thus, it is also critical to investigate microbiota and other emerging factors such as nutrients for developing novel targeted therapy to promote intestine repair.
